# The CTX-M-15-Producing *Escherichia coli* Clone O25b: H4-ST131 Has High Intestine Colonization and Urinary Tract Infection Abilities

**DOI:** 10.1371/journal.pone.0046547

**Published:** 2012-09-28

**Authors:** Sophie Vimont, Anders Boyd, Alexandre Bleibtreu, Marcelle Bens, Jean-Michel Goujon, Louis Garry, Olivier Clermont, Erick Denamur, Guillaume Arlet, Alain Vandewalle

**Affiliations:** 1 AP-HP, Hôpitaux Universitaires Est Parisien - site Tenon, Service de Bactériologie, Paris, France; 2 Université Paris 6 - Pierre et Marie Curie, Paris, France; 3 INSERM U707, Paris, France; 4 Université Paris Diderot, Sorbonne Paris Cité, Paris, France; 5 INSERM U722, Paris, France; 6 INSERM U773, Paris, France; 7 Unité de Pathologie Ultrastructurale et Expérimentale, Centre Hospitalier et Universitaire de Poitiers; Université de Poitiers, Poitiers, France; University of Würzburg, Germany

## Abstract

Increasing numbers of pyelonephritis-associated uropathogenic *Escherichia coli* (UPEC) are exhibiting high resistance to antibiotic therapy. They include a particular clonal group, the CTX-M-15-producing O25b:H4-ST131 clone, which has been shown to have a high dissemination potential. Here we show that a representative isolate of this *E. coli* clone, referred to as TN03, has enhanced metabolic capacities, acts as a potent intestine- colonizing strain, and displays the typical features of UPEC strains. In a modified streptomycin-treated mouse model of intestinal colonization where streptomycin was stopped 5 days before inoculation, we show that TN03 outcompetes the commensal *E. coli* strains K-12 MG1655, IAI1, and ED1a at days 1 and 7. Using an experimental model of ascending UTI in C3H/HeN mice, we then show that TN03 colonized the urinary tract. One week after the transurethral inoculation of the TN03 isolates, the bacterial loads in the bladder and kidneys were significantly greater than those of two other UPEC strains (CFT073 and HT7) belonging to the same B2 phylogenetic group. The differences in bacterial loads did not seem to be directly linked to differences in the inflammatory response, since the intrarenal expression of chemokines and cytokines and the number of polymorphonuclear neutrophils attracted to the site of inflammation was the same in kidneys colonized by TN03, CFT073, or HT7. Lastly, we show that *in vitro* TN03 has a high maximum growth rate in both complex (Luria-Bertani and human urine) and minimum media. In conclusion, our findings indicate that TN03 is a potent UPEC strain that colonizes the intestinal tract and may persist in the kidneys of infected hosts.

## Introduction

Urinary tract infections (UTIs) are one of the bacterial infections that most often affect children, young adults, and also renal transplanted patients. UTIs are mainly due to uropathogenic *Escherichia coli* (UPEC), which are becoming more resistant, thus hampering the therapeutic management of UTI.

*E. coli* is a ubiquitous human pathogen responsible for both community- and hospital-acquired infections. Over the past five decades, scientists have witnessed increasing antimicrobial resistance in the community setting. Initially, resistance was previously limited to certain specific antibiotics, such as ampicillin or trimethoprim [1], but recently the horizon of resistance has expanded, with the emergence of broad resistance to large families agents. In particular, plasmid-mediated extended-spectrum ß-lactamases (ESBLs), have become prominent in the community [2] [3]. Unfortunately, in addition to being resistant to most-ß-lactam antibiotics, ESBL producers are also often resistant to aminoglycosides and fluoroquinolones. Over the last decade, the CTX-M enzymes, have gradually replaced the classical TEM and SHV-type ESBLs in many countries [4], [5] and have gained worldwide attention. In 2008, two research groups, analyzing the population of *E. coli* ESBL-producing strains, described a particular CTX-M-15-producing clonal group. This clone occurred in both in-patients and out-patients worldwide, strongly suggesting that it is widely disseminated [6], [7], which constitutes a major health problem [8], [9]. Indeed, such bacterial resistance frequently delays the establishment of appropriate therapy [10], leading to higher costs and increased use of the “last resort” antimicrobials (i.e. carbapenems) [5]. This clone, which exhibits an O25b:H4 serotype, belongs to phylogenetic group B2 and sequence type (ST) 131 [7], [11]. Interestingly, several studies have confirmed the worldwide prevalence of this *E. coli* with the ST131 sequence type harboring a broad range of resistance genes on a transferable plasmid, mostly from the CTX-M family, and virulence genes [12]. This ST131 sequence type has also been detected in companion animals, non-companion animals, and food [12].

The clinical spectrum of disease induced by ST131 *E. coli* is similar to that for other *E. coli*. UTIs predominate, ranging from uncomplicated cystitis to life threatening sepsis. As this clone is mainly isolated from UTI, we determined the ability of a representative isolate of the O25b:H4 ST131 CTX-M-15 *E. coli* clone, known as the TN03 strain [11], [13], [14], first to colonize the intestine and then to infect the kidney using experimental mouse models in order to gain insights into its evolutionary success.

## Results and Discussion

### The TN03 strain is a potent colonizer of the intestine

**Figure 1:**
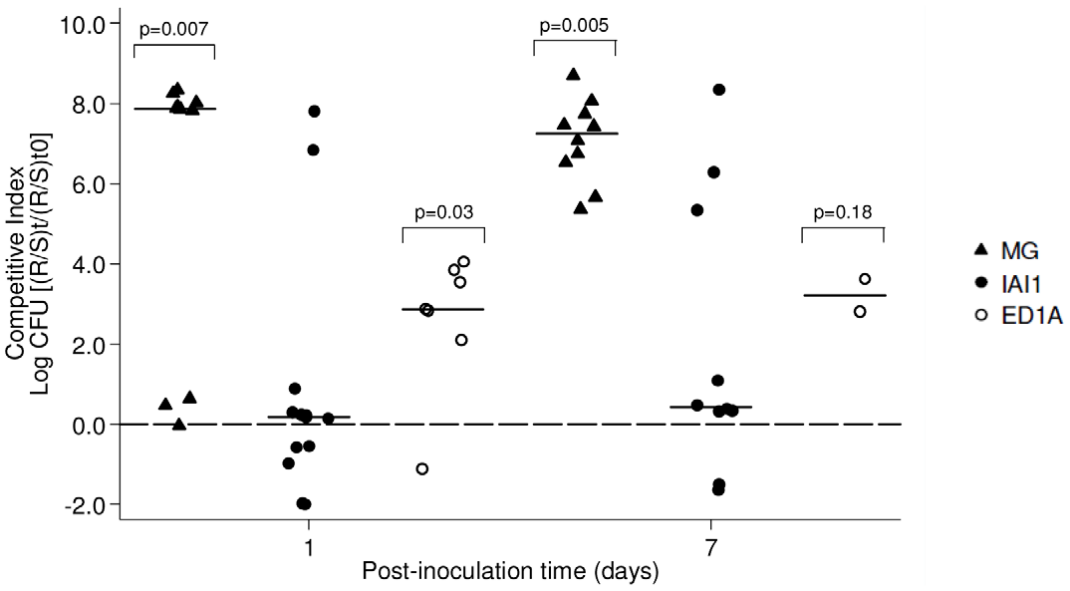
Intestinal colonization. Intestinal competition tests were performed in streptomycin-pre-treated CD1 mice. Competitive indexes (CIs) are given for day 1 and day 7 post-inoculation. The CI index is a ratio of ratios, in which the ratio between resistant (TN03) and sensitive (K-12 MG1655, IAI1 or ED1a) strains at post-inoculation time points is divided by this same ratio at the initial inoculation time. Horizontal bars represent median log_10_ CI ratios and compared to a ratio of null effect (0, that is log_10_ 1.0) using a Wilcoxon signed-rank test.

Infection of the urinary tract presumably begins with the colonization of the bowel by a uropathogenic strain [15], as suggested by the fact that the UPEC isolates present in infected urine are almost always detectable in the host's fecal flora at the time of presentation [16]. We therefore determined the ability of *E. coli* strain TN03 to colonize the gut using a mouse model of competition for intestinal colonization. A classical streptomycin-treated mouse model is usually used [17], because *E. coli* colonization cannot be studied experimentally in conventional animals due to colonization resistance. Streptomycin is used in this case to eliminate the natural coliform intestinal population and allows *E. coli* to colonize. Indeed, such experiments require an animal model with open niches where *E. coli* can colonize in relatively high numbers, but must also have a dense and diverse anaerobic community that matches the native microbiota of the conventional animal as closely as possible. In our case, however, the TN03 strain is resistant to streptomycin in contrast to all its other experimental competitors. Although, *in-vitro* streptomycin competitor mutants could have been used, these mutations could have been a burden on the fitness of the strains, which would have introduced bias into the experiment. We decided to modify slightly the classical protocol. Six-week-old CD1 female mice were pre-treated with streptomycin (5 g/liter) during five days in order to create the appropriate conditions for colonization. Streptomycin administration was then stopped five days before inoculation, thus avoiding bias linked to antibiotic pressure. Natural coliform removal efficiency was checked just before bacterial inoculation. The absence of residual streptomycin in the stools was assessed by a microbiological assay (data not shown). Mice were given 10^6^ of each *E. coli* strain *per os*: the TN03 bacteria, or one of three commensal strains, belonging to different phylogenetic groups: the K-12 MG1655 [18], IAI1 and ED1a strains [19] from phylogenetic groups A, B1 and B2, respectively (Table 1), to check the individual capacities of each strain to colonize the mouse intestinal tract. The sizes of bacterial populations in the intestine were evaluated at days 1 and 7 following administration. All individual strains colonized the mouse intestine at levels ranging from 10^4^ to 10^9^ bacteria/g feces at day 1, and from 10^3^ to 10^9^ bacteria/g feces at day 7, with no difference being found between the strains (data not shown). The observed intra-strain variability can be attributed to the variability of the natural mouse microbiota in the absence of streptomycin treatment. To circumvent this variability, we decided to evaluate the competition capacities of the pathogenic *E. coli* TN03 isolate. To do this, the bacteria were mixed in a 1∶1 ratio of TN03 with commensal *E. coli* K-12 MG1655, IAI1, or ED1a bacteria. 10^6^ of this bacterial mix was then administered *per os* to the mice as described above, The sizes of the bacterial populations in the intestine were evaluated at days 1, 2, 4 and 7 following inoculation. They tended to decrease slowly over time (data not shown). A competitive index (CI) which could be used to compare the colonization success of the different strains was calculated as previously described [20]. Analysis of the CI showed that the TN03 *E. coli* strain had outcompeted all of the commensal *E. coli* strains. Significant differences between TN03 with K-12 MG1655 or ED1a as a competitor (CI_MG-D1_ = 7.87, *p = 0.007*, CI_ED-D1_ = 2.86, *p = 0.03*) were already detected day 1 post-bacterial administration, and persisted between TN03 with K-12 MG1655 (CI_MG-D7_ = 7.25, *p = 0.005*) at day 7 post-bacterial administration (Fig. 1). It is worth noting that all the competitors did not react equally strongly, and that it was easier for TN03 to compete with K-12 MG1655 or ED1a than with IAI1, although TN03 was always the most effective. These data attested that the TN03 strain has a high capability to colonize the intestine rapidly, for a period of at least one week, despite the presence of competitors.

**Table 1:** Main characteristics of the *E. coli* isolates used in this study.

Strain ID	K-12 MG1655	IAI1	ED1a	TN03	HT7	CFT073
**Origin**	Feces	Feces	Feces	Urine	Urine	Blood
**Pathogenicity**	Commensal	Commensal	Commensal	UTI	UTI	Urosepsis
**Phylogenetic group** 1	A	B1	B2	B2	B2	B2
**ST Achtman** 2	98	1128	452	131	95	73
**B2 sub-group** 3	/	/	VIII	I	IX	II
**O-type**	O16	O8w	O81	O25b	O1	O6
**Virulence factors**						
Adhesins						
*fimH*	+	+	+	+	+	+
*papC*	−	−	−	−	+	+
*papG*	−	−	−	−	II	II
*sfa/foc*	−	−	−	−	−	+
*hra*	−	−	−	−	−	−
*iha*	−	−	+	+	−	+
*ibeA*	−	−	−	−	−	−
Toxins						
*hly*	−	−	−	−	−	+
*sat*	−	+	−	+	−	+
*cnf1*	−	−	−	−	−	−
Siderophores						
*iroN*	−	−	−	−	+	+
*aer (iucC)*	−	−	+	+	+	+
*fyuA*	−	−	+	+	+	+
*irp2*	−	−	+	+	+	+
*ireA*	−	−	+	−	+	+
Capsule						
*neuC-K1*	−	−	−	−	+	−
Miscellaneous						
*usp*	−	−	+	+	+	+
*ompT*	+	−	+	+	+	+
*traT*	−	−	−	+	+	−

1 Determined by the triplex PCR method of Clermont *et al.* [53].

2 According to Wirth *et al.* [55].

3 According to Le Gall *et al.* [33].

### Metabolic capacities of the TN03 strain

**Figure 2:**
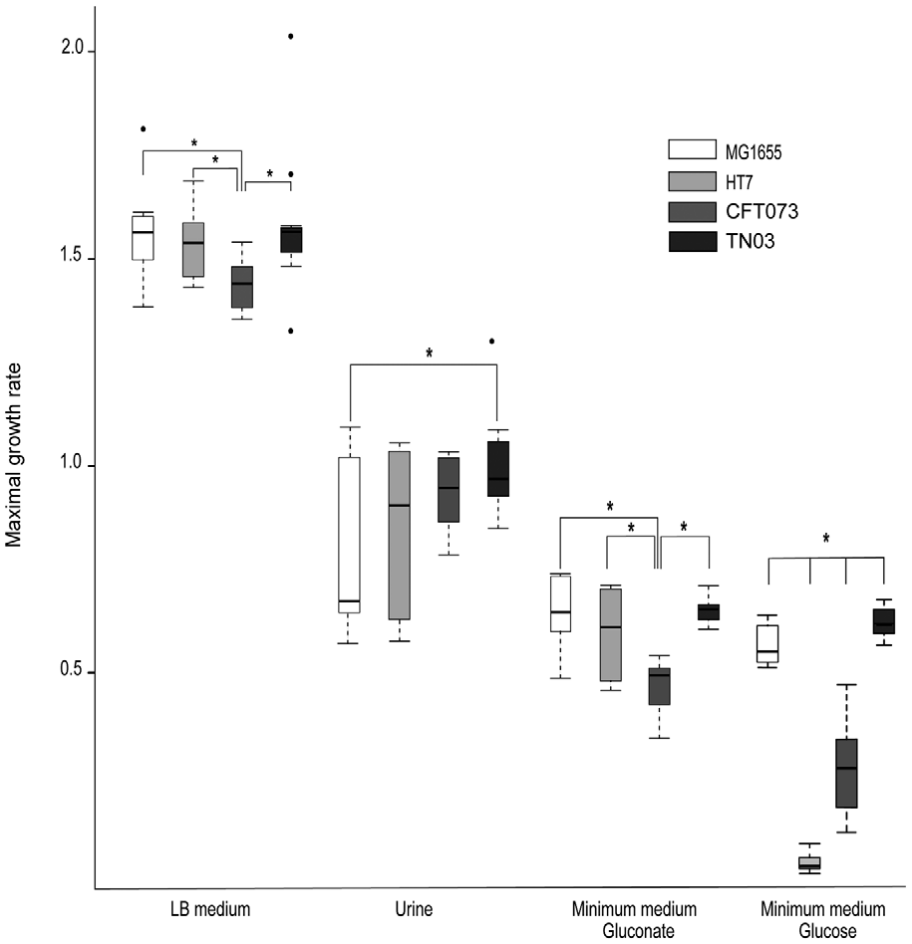
Maximum growth rate in four media. Four *E. coli* strains: K-12 MG1655 (white), HT7 (light grey), CFT073 (grey) and TN03 (dark grey) were grown in four different media: Luria Bertani (LB), urine, minimum medium with gluconate and minimum medium with glucose. Boxplots represent distribution of maximal growth rates (MGRs) calculated during the three repetitions of culture assays with the smooth spline function from R network. Black bars inside each boxplot represent medians. Dots located far from the box represent outliers. Links between boxplots with asterisks represent significant differences between two strains tested by Welch test for observed mean comparison. * p<0.05.

In order to better understand the capacity of this strain to colonize the intestinal tract, we first checked for potential production of bacteriocins by TN03. These antimicrobial peptides are widespread among the Bacteria and Archaea and are active against other bacteria, whether of the same species or across genera [21]. TN03 strain of *E. coli* was spotted onto a layer of *E. coli* K-12 MG1655 spread on a mitomycin plate and incubated for 18 h at 37°C. No halo was visible around the spot, indicating that no colicin (or phage) was present. Moreover, TN03 appears to be devoid of the *iroN* gene [11] (Table 1), which is associated with the main microcin genes (*micH47* and *micV*) [22]. Given these negative results, we then checked whether the TN03 strain had better metabolic capacities than the other strains. This experiment was based on the hypothesis that *E. coli* is probably a highly successful competitor in the mammalian colon, because it exploits its ability to utilize the available nutrients more efficiently than other resident species [15]. We therefore studied the bacterial metabolic capacities of the TN03 strain by measuring its maximal growth rate (MGR) under three culture conditions using Luria Bertani (LB) broth, a rich medium, and two minimum media: a minimum medium containing gluconate (MMGluconate), as gluconate has been reported to be a major carbon source in the intestine [23], and a minimum medium containing glucose (MMGlc). Indeed, in addition to the capacity of TN03 to colonize the bowel, the question arises as to whether the TN03 strains exhibit better metabolic capacities than other UPEC strains. We therefore compared the metabolic capacity of TN03 to that of the non-pathogenic K-12 MG1655 strain and two B2-UPEC strains, *i.e.* CFT073 [24] and HT7 [25]. All the strains studied were precultured in the corresponding medium for 24 h with constant stirring. They were each then inoculated into the appropriate medium into plates containing 96 flat-bottomed wells. OD_600_ was measured every 5 minutes in order to determine the growth over a period of 24 hours. Interestingly, the TN03 strain exhibited high *in-vitro* growth capacity in all three media tested (Fig. 2). TN03 had a greater MGR than CFT073 in LB (1.58 *vs* 1.43, p = 0.01), or MMGluconate (0.65 *vs* 0.47 *p<0.01*). TN03 also exhibited a higher MGR that the other three strains (TN03-K-12 MG1655 0.64 vs 0.55 p = 0.01; TN03-HT7 0.64 vs 0.06 p<0.01; TN03-CFT073 0.64 vs 0.31 p<0.01) when grown in MMGlc. Taken together, these data suggest that TN03 has high metabolic potential, facilitating its adaptation to different environments, and probably an enhanced ability to establish and maintain intestinal colonization, the first step of uropathogenicity. These data support the hypothesis suggested by Johnson *et al* [26] that ST131 strains may have enhanced fitness for upstream steps, including colonization.

### The TN03 strain is able to infect the urinary tract for at least 7 days

**Figure 3:**
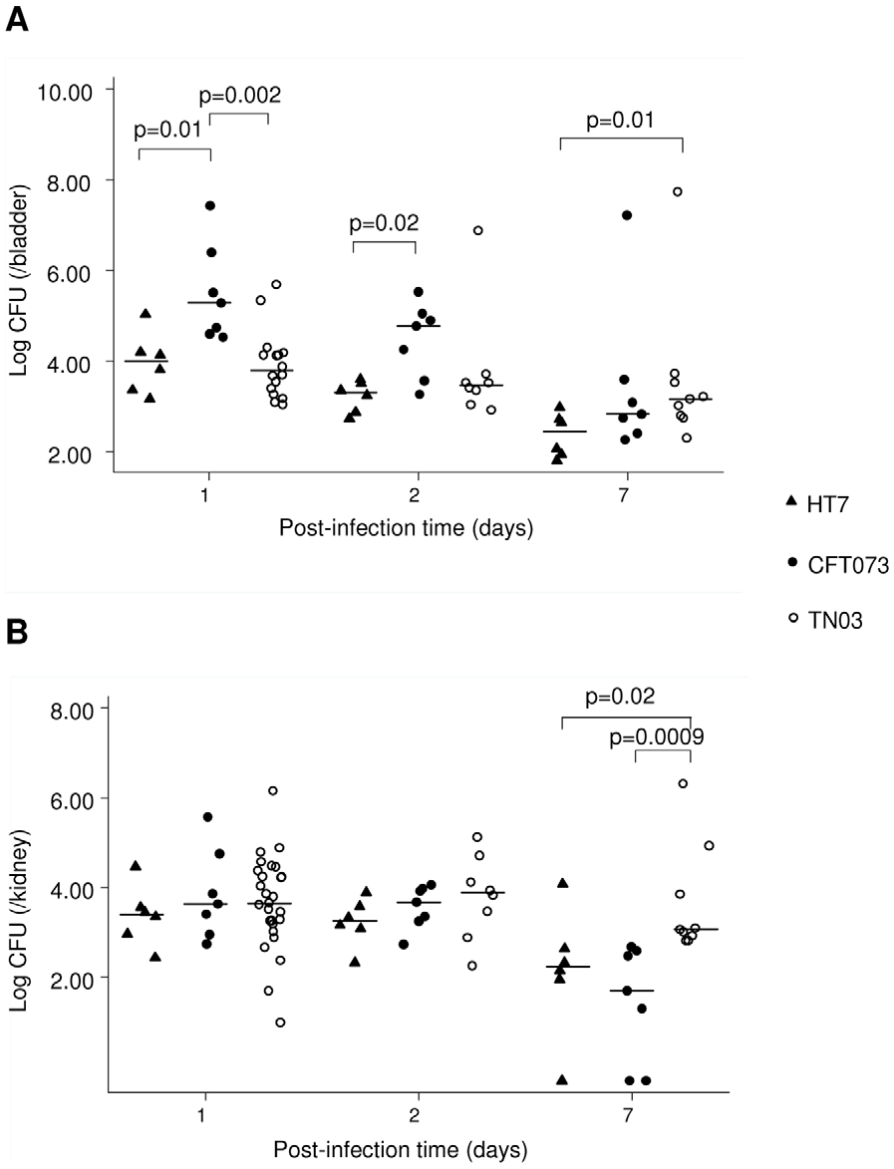
Experimental model of UTI. C3H/HeN mice were infected with the HT7, CFT073, or TN03 strains directly by transurethral inoculation of the bacteria (10^8^ cfu in 50 µl sterile PBS). At day 1, day 2 and day 7 post infection, bladders (A) and kidneys (B) were harvested and their bacterial burdens determined. Horizontal bars represent median log_10_ bacterial counts. Statistical differences between strains at each post-infection time point were performed using the Kruskal-Wallis equality-of-populations rank test.

After having colonized the intestinal bowel, UPEC are then able to colonize the periurethral area, and secondly colonize the lower (*i.e.* the bladder) and the upper (*i.e.* kidneys) urinary tract. To better understand the uropathogenic abilities of the *E. coli* TN03 strain, experiments were then performed using an experimental model of ascending UTI as previously described [25], [27]. Renal retrograde urinary tract infection was carried out on C3H/HeN (Lps^n^) mice, which were then sacrificed at days 1, 2, and 7 post bacterial inoculation. Median log_10_ bacterial counts per bladder or per kidney were calculated and compared in the different strains at each of the post-infection time-points (days 1, 2, and 7) using a Kruskal-Wallis equality-of-populations rank test. Murine models of UTI have been established in different strains of mice with different genetic backgrounds, such as C57BL/6, CBA, FVB/NJ or different strains of C3H mice (for review see [28]). However, these strains exhibit large differences in the levels of bacterial loads in the lower and upper urinary tract and in the degree of host inflammatory response. Whereas C57BL/6 mice produce a more robust inflammatory response and resolve acute infection and bacteriuria more rapidly than the other strains, C3H background mouse strains have been reported to develop chronic UTI for up to 2 weeks post-infection [29]–[31]. Studies using CH3/HeN (LPS^n^) mice exhibiting functional Toll-like receptor 4 (TLR4), and C3H/HeJ (LPS^d^) mice exhibiting an inactivating mutation in the *tlr4* gene have demonstrated the importance of the UPEC-mediated TLR4 signaling pathway (reviewed in [32]). We also showed that different UPEC strains colonize the kidneys of C3H/HeN mice [25]. C3H/HeN mice were therefore used as a model of chronic infection to study the outcome of the early and delayed kinetics of renal colonization and renal inflammatory response caused by TN03, which were compared, as controls, to the same two B2-UPEC strains used above: CFT073 and HT7. These strains belong to B2 subgroups II (ST73) and IX (ST95), respectively, whereas TN03 belongs to subgroup I (ST131) (Table 1) [33]. As CFT073 is cytotoxic for renal tubule epithelial cells, whereas HT7 is not [25], mice were infected either with the TN03 strain or with the CFT073 or the HT7 strains. Like the UPEC CFT073 and HT7 strains [34], [35], the *E. coli* TN03 strain colonized the bladder and the kidneys 24 h after retrograde inoculation. One and two days after the inoculation of UPEC, the bacterial burdens were significantly greater in the bladders from CFT073-infected mice than those from HT7- or TN03-infected mice, but no significant difference was observed in the bacterial loads at days 1 and 2 in the kidneys (Fig. 3 A and B). However, at day 7, the median value of CFU in the bladder was greater for TN03 than for HT7 (1480 *vs* 283 CFU/bladder, p = 0.01), and to a lesser extent, for CFT073 (Fig. 3A). In the kidneys, the median value of CFU at day 7 was significantly higher for TN03 than for HT7 (1170 *vs* 175 CFU/kidney, p = 0.02) or CFT073 (1170 *vs* 50 CFU/kidney, p = 0.0009) isolates (Fig. 3B). These data, which highlighted the better capacities of this strain to persist in the kidneys, led us to check its metabolic capacities in the urinary tract. We therefore compared the MGR of the TN03 strain, the CFT073 and HT7 UPEC strains and the K-12 MG1655 strain in urine from healthy volunteers as described above. Interestingly, the TN03 strain grew better than K-12 MG1655 (0.98 *vs* 0.81, p = 0.03), and to a lesser extent, better than the other UPEC strains analyzed (Fig. 2). Collectively, these findings suggest that the TN03 strain is indeed an UPEC strain, in agreement with the study of Totsika *et al.* [36] on ST131 *E. coli* strains. We showed, in addition, that the TN03 UPEC strain invades the bladder less rapidly than the CFT073 strain, but persists at higher level in the bladder and kidneys than the UPEC CFT073 and HT7 strains, one week after bacterial inoculation.

### TN03 induces a low renal host inflammatory response

**Figure 4:**
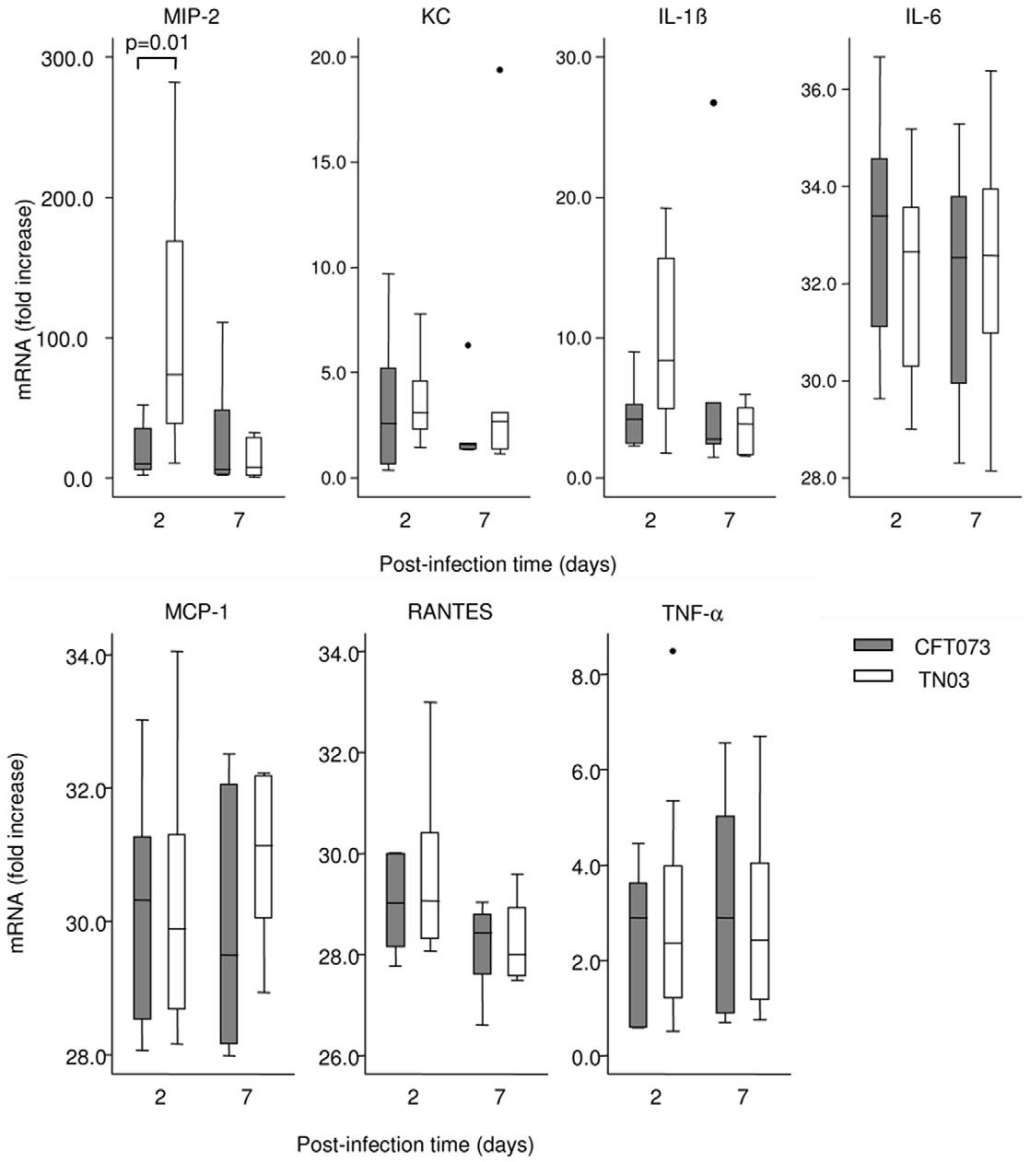
Expression of pro-inflammatory mediators in kidneys infected by UPEC. Expression of MIP-2, KC, IL-1ß, IL-6, MCP-1, RANTES and TNF-α in the day 2 and day 7 post-infection kidneys following the transurethral inoculation of the CFT073 and TN03 UPEC isolates. mRNA expression of each inflammatory marker was calculated using the −ΔΔCT method. Values are expressed as the relative fold increase in kidneys of each mRNA level of proinflammatory mediator in comparison with that measured in naive mice. Horizontal bars represent the median values. Statistical differences between the strains were determined at each time point using the Kruskal-Wallis equality-of-populations rank test.

We next investigated the host's inflammatory response following urinary tract infection caused by TN03. For this purpose, we analyzed the levels of mRNA expression of the TNF-α cytokine and 6 pro-inflammatory cytokines, such as MIP-2, KC, IL-1ß, RANTES, IL-6, and MCP-1, by real-time PCR as previously described [25] in kidneys from mice infected with TN03 using CFT073 UPEC as a control. Among the proinflammatory mediators tested, only MIP-2/CXCL2, which is a chemoattractant chemokine playing a key role in the migration of polymorphonuclear neutrophils to the site of inflammation [37], [38], showed a significantly higher level in the day-2 TN03 than day-2 CFT073 post-infected kidneys (Fig. 4). The expression level of KC/CXCL1 which also plays a key role in the neutrophils chemoattraction and the expression levels of the other pro-inflammatory mediators tested (Fig. 4), as well as the number of Ly6-G-positive neutrophils attracted to the kidneys, did not differ significantly either in the day 2 post TN03-infection kidneys versus the CFT073-infected kidneys nor in those for day 7 (data not shown). These findings suggest that TN03 causes quite similar inflammatory response compared to that induced by CFT073. Colonization of the urinary tract by UPEC induces the rapid (within the first 6–12 h) activation of proinflammatory mediators and the recruitment of the neutrophils for the efficient phagocytic killing of bacteria. Thereafter, the increase in pro-inflammatory mediators tends to return to normal levels. Given that all measurements were performed 48 h after the inoculation of UPEC, no direct conclusions could be drawn concerning the possibility that TN03 alters the inflammatory response and subsequent recruitment of neutrophils to the site of inflammation. Bacterial attachment to mucosal bladder and renal epithelial cells by fimbrial adhesins constitutes the initial step in UPEC pathogenicity. Pyelonephritis-associated UPEC strains usually express P-fimbriae, such as PapGII, which preferentially binds to the globoseries glycosphingolipids that are abundantly expressed on the surface of renal epithelial cells (see for review [39]). The attachment of fimbriated UPEC to renal epithelial cells triggers the innate host response, mediated in part by TLR4, which recognizes lipopolysaccharide [27], [40]–[42]. The activation of TLR4 signaling stimulates the production of pro-inflammatory mediators leading to the recruitment of neutrophils and macrophages to the site of inflammation for the efficient killing of bacteria. The fact that TN03 does not express PapGII (Table 1), could suggest that the low binding capacity of TN03 to epithelial cells may favor the persistence of long-lived bacteria within the kidneys. The ability of the UPEC TN03 strain to limit the inflammatory response and the subsequent clearance of bacteria, and to persist in the upper urinary tract without activating a significant inflammatory response calls for further studies.

### Concluding remarks

Special pathogenicity traits and the prevalence of the *E. coli* strain in the fecal microbiota are both important features required for the occurrence of UTI [16]. Virulence traits and group B2 status were shown to be closely associated with the fecal abundance and dominance of *E. coli* strains and their pauciclonality – *i.e.* the fact that no more than 4 different *E. coli* clones coexist concurrently. Collectively, these various different factors contribute jointly to UTI pathogenesis, in association with other still-unidentified virulence factors and other group B2-associated characteristics. This latter may promote intestinal dominance [33], thereby increasing the probability that the subsequent steps in pathogenesis will occur. Johnson *et al* [26] also suggest that the epidemiological success of ST131 may reflect the fact that it displays greater fitness during the upstream steps involved in pathogenesis, colonization or in transmission. In this study, we suggest that the TN03 strain has higher metabolic capacities, and that these may explain its enhanced capacities to colonize and subsequently to cause infectious disease. Indeed, epidemiological studies both during UTI in 2003 [43] and on stools of healthy subjects in 2006 [44] have reported a high prevalence of the ST131 clone of *E. coli* (3% and 7%, respectively). Rogers *et al* [12] and Johnson *et al* [45] also confirmed the worldwide prevalence of ST131 harboring a broad range of virulence and resistance genes. Undoubtedly, the increasing prevalence of the ESBL not only at the hospital, but also in the community setting, suggests that this particular clone harboring ESBL is also increasing. However, further investigations are needed to define the current prevalence of this clone more precisely.

The spectrum of clinical infections caused by the ST131 clone appears broadly similar to that of other strains of *E. coli*. The urinary tract is the most common site of human infection at which *E. coli* predominates. Pitout *et al* [46] identified a greater propensity for urinary sepsis above other sites of infection in ST131 than in non-ST131 *E. coli* bacteriemia. The results from the present work suggest that the specific TN03 ST131 strain expressing CTX-M is a potent uropathogenic strain that can efficiently colonize the intestine, as a result of its greater metabolic capacities or other still-unknown factors, and subsequently infect the urinary tract, conferring on it an intrinsic evolutionary success, regardless of its selection as a result of antibiotic pressure. This TN03 clone possesses a number of virulence factors (Table 1), including the pathogenicity island HPI (*fyuA*, *irp2*), siderophores (*iutA*, *iha*), the capacity for serum resistance (*traT*) and a capsule (*kpsE*) [47], but it does not possess the classical extraintestinal PAIs (PAI_CFT073_, PAI II_J96_ and PAI III_536_), which may limit the inflammatory host response and the subsequent clearance of bacteria. The group of Frimodt-Møller showed that some B2 *E. coli* strains, with a higher aggregate virulence factor genes score, are associated with persistence or relapse in recurrent UTI [48]. In this line, TN03 also has the ability to form a biofilm [11], a property which may contribute to its long-term persistence in various environments and to its resistance to antimicrobial agents or disinfectants [49].

However, our data should be balanced by the fact that we have studied only one isolate representing the ST131 clone, *i.e.* TN03, and that high genetic [45], [50], [51] and intrinsic virulence [26] polymorphism has been reported within isolates of this clone. More isolates clearly need to be studied.

Given the rapid emergence of the ST131 CTX-M-15 *E. coli* clone, particular attention should be paid to this particular uropathogenic strain, which expresses resistance to classical antibiotic therapy and constitutes a major health problem [8]. Our experimental data on a representative strain of the emerging O25b ST131 clone support the link between the B2 group, intestinal colonization, virulence determinants, and UTI. Further studies will be required to identify the additional factor(s) responsible for intestinal colonization and the ability of the TN03 strain to invade the urinary tract system.

## Materials and Methods

### Bacterial strains and growth conditions

Experiments were carried out using several different *E. coli* strains: the TN03 strain, which is a representative isolate of the O25b:H4-ST131 CTX-M-15 *E. coli* clone [11], two UPEC strains belonging to phylogenetic group B2: the CFT073 strain [24] and the HT7 strain, which was isolated from the infected urine of a young woman hospitalized following an episode of pyelonephritis at the Tenon hospital (Paris, France) [27], [35], two commensal *E. coli* strains: IAI1 and ED1a, one from phylogenetic group B1 and the other from phylogenetic group B2, and the non-pathogenic laboratory strain *E. coli* K-12 MG1655 [18] (Table 1). When not otherwise indicated, bacteria were grown in LB broth with shaking for 18 h at 37°C.

For the comparative growth assays, strains were grown at 37°C in four different media: LB (tryptone 10 g, yeast extract 5 g, NaCl 10 g/L), minimum medium with glucose (MMGlc) 20 mM [NaH_2_PO_4_,1 H_2_O 4.69 g, Na_2_HPO_4_ 11.15 g/L, (NH_4_)_2_SO_4_ 2.65 g/L, MgSO_4_ 7 H_2_O 0.075 g/L, KCl 3 g/L, FeCl_3_ 0.2 ml Molar Solution 0.1%, glucose 20 mM/L], minimum medium with gluconate (MMGluconate) 20 mM (the same as the minimum medium, but with glucose instead of gluconate), and urine. Urine was collected from ten healthy male volunteers who were not taking any medication, pooled, filtered, and stored at −20°C before use. LB and urine are complex media, whereas MMGlc and MMGluconate are minimal media with only one source of carbon. The MMGluconate is used, as this sugar has been reported to be a major source of carbon in the intestine [23]. All the strains studied were grown overnight (O/N) in the three media in flasks at 37°C with constant stirring at 280 rpm. O/N cultures were prediluted at 1/100 in PBS and strains were inoculated in twelve different wells each at 1/100 in a Costar® 96 flat-bottomed well plate. Growth was recorded by an Infinite 200 Tecan®, which measured the OD_600_ in each well every 5 minutes at 37°C, while stirring for 24 hours. Growth assays were repeated 3 times. The MGR was calculated from growth curves obtained by Tecan®. OD_600_ in nm were collected and Log-transformed. Curves were calculated from a smoothed spline function. The MGR was defined as the time point at which the maximum value of the derivative of the smoothed function was observed. All MGRs were compared by strain and by medium using a Welch test.

### Intestinal colonization

Six-week-old female mice (Charles River CD-1) pretreated with streptomycin were used to monitor the ability of *E. coli* strains to colonize the intestine of a mammalian host. The mice were isolated and had free access to sterile food and drinking water supplemented with streptomycin sulfate (5 g/liter) for 5 days. The mice did not receive streptomycin for 5 days before bacterial inoculation. This modification of the classical streptomycin-treated mouse colonization assay [17] permits the subsequent colonization of the mouse intestine by streptomycin-sensitive strains. The effectiveness of the antibiotic treatment against the coliform intestinal population was checked by plating a pure suspension of feces on Drigalski's selective agar medium. The absence of streptomycin was also confirmed by assaying the antibiotic in the stools. The day of inoculation, 10^6^ *E. coli* bacteria were then administered in 200 µl of PBS by oral route to mice free of coliform flora, either alone or mixed at a ratio of 1∶1. On days 1, 2, 4 and 7 after bacterial administration, the sizes of bacterial populations in the intestine of mice were evaluated by plating dilutions of weighed fresh feces on Drigalski's agar with or without 2 µg/ml cefotaxime. At least four mice were used in three independent experiments. As previously described [20], a competitive index (CI) was calculated. The CI index is a ratio of ratios, in which the ratio between resistant and sensitive strains at post-inoculation time points is divided by the same ratio at the initial inoculation time. All experiments were performed in accordance with the recommendations of the French Ministry of Agriculture and approved by the French Veterinary Services (accreditation number A 75-18-05).

### Production of colicins and phages

Colicins and phages were detected as previously described [52]. Briefly, they were detected using an overnight (O/N) preculture suspension of *E. coli* K-12 MG1655 as a sensitive strain. K-12 MG1655 bacteria were plated on a LB agar medium containing mitomycin (25 µm/L). Then, 10 µl of an O/N culture in LB medium of each strain were spotted. After an O/N culture at 37°C, the presence of colicin (or phage) were detected for the strains surrounded by a halo reflecting an inhibition of the culture of the *E. coli* K-12 strain.

### Bacterial determinants

As previously described, the strains were assigned to 1 of the 4 main *E. coli* phylogenetic groups, i.e., groups A, B1, B2, and D, using triplex PCR developed previously by Clermont et al [53]. MLST was performed by gene amplification and sequencing as in [54] and/or [55]. The B2 subgroup O-type was determined as described by Le Gall *et al* [33]. The presence of 19 virulence factors representative of identified *E. coli* extraintestinal virulence determinants were tested by PCR [56].

### Murine model of ascending urinary tract infection

Renal retrograde urinary tract infection was carried out on 8-week-old female C3H/HeN (Lps^n^) mice (Janvier Breeding Center, Le Genest St Isle, France), which were caged individually. Mice subjected to water restriction for 12 h were anesthetized, and then infected with various *E. coli* strains introduced (10^8^ bacteria diluted in 50 µ l sterile PBS) *via* the transurethral route into the bladder as described previously [27], [35]. Once the mice had recovered from anesthesia, they were provided with water for the 7 days following the bacterial inoculation. Mice were then sacrificed at days 1, 2, and 7 after bacterial inoculation, and the kidneys and bladder were aseptically removed. Two halves of one kidney were fixed or quickly frozen in liquid nitrogen, and the contralateral kidney was homogenized, diluted in sterile PBS, and plated on LB agar plates to count the number of colony forming units (CFUs). Kidney sections were stained using an anti-Ly6-G antibody (BD Biosciences France SA) to quantify the number of infiltrating neutrophils. Mice were infected either with the TN03 strain or with either the UPEC CFT073 or HT7 strain belonging to phylogenetic group B2. At least ten mice were used for each condition. The experiments were performed in accordance with the guidelines of the French Agriculture Ministry (see above).

### Quantitative real time PCR

Total RNA was extracted from kidneys using the RNABle kit (Eurobio, Courtaboeuf, France), and reverse-transcribed using Moloney Murine Leukemia Virus reverse transcriptase (Invitrogen, Cergy-Pontoise, France). cDNA was subjected to real-time PCR, using a Chromo IV sequence detector (MJ Research, Waltham, MA) using mouse primers and TaqMan probes used for 6 different proinflammatory cytokines: chemokine (C-X-C motif) ligand 2 (CXCL2) also called macrophage inflammatory protein 2 α (MIP-2α), chemokine (C-X-C motif) ligand 1 (CXCL1) also called keratinocyte-derived chemokine (KC), Interleukin-1β (IL-1β), Interleukin-6 (IL-6), chemokine (C-C motif) ligand 2 (CCL2) also known as monocyte chemoattractant protein-1 (MCP-1), chemokine (C-C motif) ligand 5 (CCL5) also known as regulated upon activation normal T cell expressed and secreted (RANTES); the tumor necrosis factor-α (TNF-α) cytokine, and ß-actin as previously described earlier [25]. PCR data were reported as the relative increase, after infection, in mRNA transcripts *versus* non infected kidneys, and corrected by the levels of ß-actin mRNA, used as the internal standard. Values are the means of 6–8 separate determinations.

### Statistical analysis

The values are given as medians (IQR) and, unless specified otherwise, comparisons between strains were performed using either the Wilcoxon signed-rank test or the Kruskal-Wallis equality-of-populations rank test. All statistics were computed using STATA (v10.0, College Station, TX, USA) or R (R Development Core Team, 2009, Vienna, Austria) and statistical significance was determined at a p-value of less than 0.05.
